# Circulating Galectin-3 Evaluation in Dogs With Cardiac and Non-cardiac Diseases

**DOI:** 10.3389/fvets.2021.741210

**Published:** 2021-10-14

**Authors:** Ga-Won Lee, Min-Hee Kang, Woong-Bin Ro, Doo-Won Song, Hee-Myung Park

**Affiliations:** Laboratory of Veterinary Internal Medicine, College of Veterinary Medicine, Konkuk University, Seoul, South Korea

**Keywords:** dog, galectin-3, fibrosis, heart disease, cardiomyopathy

## Abstract

Galectin-3 is involved in important biological functions such as fibrogenesis and inflammation. Notably, it is associated with various diseases and plays a major role in cardiac inflammation and fibrosis. Although heart diseases are relatively common in dogs, a few studies have analyzed the circulating galectin-3 concentration in dogs with various heart diseases, including myxomatous mitral valve disease, patent ductus arteriosus, and pulmonic stenosis. The aims of the present study were to evaluate the effect of heart disease on circulating galectin-3 levels in dogs, and also to evaluate the correlation between galectin-3 concentration and conventional echocardiographic indices along with N-terminal pro-B-type natriuretic peptide (NT-proBNP) concentration in dogs with heart diseases. The medical records and archived serum samples of 107 dogs were evaluated retrospectively. In total, 107 dogs were classified into healthy dogs (*n* = 8), cardiac disease (*n* = 26), and non-cardiac disease groups (*n* = 73). The circulatory galectin-3 levels were analyzed using a commercially available canine-specific galectin-3 enzyme-linked immunosorbent assay kit. This study demonstrated that dogs with heart, endocrine, and dermatologic diseases had significantly higher galectin-3 levels than healthy dogs (*p* = 0.009, *p* = 0.007, and *p* = 0.026, respectively). Among dogs with heart diseases, dogs with concentric cardiomyopathy had significantly increased circulatory galectin-3 levels compared with healthy dogs (*p* = 0.028). E′/A′ had a positive association with galectin-3 levels among conventional echocardiographic indices. Moreover, the galectin-3 concentration could predict diastolic dysfunction. In dogs with myxomatous mitral valve disease, a significantly positive correlation was revealed between galectin-3 levels and NT-proBNP levels (*p* = 0.007). Overall, this study demonstrates that circulatory galectin-3 levels increase in dogs with heart, endocrine, and dermatologic diseases. Moreover, this study demonstrates that galectin-3 concentration could be helpful to evaluate cardiac remodeling and diastolic function. Further large-scale research is required to evaluate the role of circulating galectin-3 in dogs with heart diseases.

## Introduction

Galectin-3 is a 32 to 35-kDa protein that belongs to 14 members of the β-galactoside-binding lectin family, which has important biological functions such as fibrosis formation and inflammation (1–3). Galectin-3 was first revealed by Ho and Springer as a macrophage subpopulation specific marker and has since emerged as a novel biomarker (4). It is encoded by a single gene named LGALS3 and can be applied to inflammation, tissue repair, and cardiac ventricular remodeling by promoting fibroblasts to change to myofibroblasts and increasing collagen synthesis (5–7).

Galectin-3 has been reported to have an association with a variety of diseases involving the heart, liver, and kidneys, and even in cancer (3, 8). Several studies in humans have reported that the elevation of serum galectin-3 levels was significantly associated with gastric cancer (9), renal failure (10, 11), diabetes mellitus (DM) (8), and heart failure (HF) (12, 13). Furthermore, galectin-3 has a significant role in cardiac inflammation and fibrosis (1, 14). Galectin-3 is considered a protein involved in the pathogenesis of heart dysfunction (15, 16). Galectin-3 is detected in the nucleus, cytoplasm, and extracellular compartment and has been shown to participate in tissue fibrogenesis and a prognostic indicator in HF (17, 18). Moreover, it is known as a prominent prognostic biomarker for predicting the incidence of HF and cardiovascular mortality in humans (13, 19, 20).

Cardiovascular diseases are relatively common and can have systemic effects and various complications in veterinary medicine (21). The early and accurate diagnosis of heart disease is important because it worsens without treatment, and treatment options vary according to diagnosis. However, a few studies on the relationship between the circulating galectin-3 levels and various heart diseases have been conducted in veterinary medicine. One report showed increased galectin-3 concentrations in the plasma and cardiac tissues of dogs with myxomatous mitral valve disease (MMVD) (7).

B-type natriuretic peptide (BNP) is known as a remarkable biomarker for assessing cardiac function, and N-terminal pro-B-type natriuretic peptide (NT-proBNP) can be applied in clinical examinations for diagnosing heart diseases and evaluating the prognosis of patients with cardiovascular diseases (22–24). NT-proBNP is secreted when the ventricles stretch following elevation of ventricular blood volume (25). Thus, it has an ability to differentiate heart diseases from non-cardiac diseases, to evaluate the severity of heart diseases, and to provide prognosis in several heart diseases such as dilated cardiomyopathy (DCM) and MMVD (26, 27). To date, however, research on the correlation between NT-proBNP and galectin-3 levels remains scarce in veterinary medicine.

This study had the following aims: (i) to evaluate the circulatory galectin-3 levels in dogs with various diseases including cardiac diseases, (ii) to identify the difference between galectin-3 levels according to the type of heart disease or cardiac hypertrophy type, and (iii) to analyze the correlation between galectin-3 levels and conventional echocardiographic indices with NT-proBNP levels.

## Materials and Methods

### Case Selection Criteria

The stored serum samples and medical records of 99 dogs that visited the Veterinary Medical Teaching Hospital of Konkuk University between October 2012 and December 2018 were retrospectively used. The healthy dogs comprised eight apparently healthy Beagle dogs. Stored serum samples and medical data of these healthy dogs were used from a previous study, which had been approved by the Institutional Animal Care and Use Committee (R0006046 and KBNP 18-01-01).

Inclusion criteria for dogs with various diseases included a complete medical record and sufficient amount of archived serum to measure galectin-3 concentration. In case of heart diseases among various heart diseases such as MMVD, patent ductus arteriosus (PDA), pulmonic stenosis (PS), and left ventricle hypertrophy (LVH) by non-cardiac causes were included. Dogs with heart diseases were treated with furosemide (*n* = 25), ramipril (*n* = 20), pimobendan (*n* = 14), spironolactone (*n* = 10), sildenafil (*n* = 10), amlodipine (*n* = 5), hydralazine (*n* = 3), atenolol (*n* = 3), torsemide (*n* = 2), and enalapril (*n* = 2). In case of non-cardiac diseases, urologic diseases included chronic kidney disease, cystitis, urinary calculi, and renal cyst. Neoplastic diseases included mast cell tumor, lymphoma, and nasal adenocarcinoma, and neurologic diseases included only meningoencephalitis of unknown origin. Immune-mediated diseases included immune-mediated hemolytic anemia, immune-mediated thrombocytopenia, Evan's syndrome, and immune-mediated polyarthritis. Endocrine diseases included DM, hyperadrenocorticism (HAC), hypoadrenocorticism, hypothyroidism, and hypoaldosteronism, and dermatologic diseases included only atopic dermatitis (AD). The diagnosis of each disease was based on clinical symptoms and diagnostic test results. Dogs with previously unmentioned disease and multiorgan diseases were excluded from this study.

In dogs with MMVD, the diagnosis and classification of MMVD were based on the American College of Veterinary Internal Medicine (ACVIM) cardiac disease guidelines (28). This classification system provides a continuum of stages, with class A denoting high risk for heart disease and class D representing end-stage congestive heart failure (CHF). Class B includes animals with structural heart disease but no clinical signs of CHF. In class B1, cardiac remodeling is absent on radiographic or echocardiographic findings. In contrast, radiographic and echocardiographic cardiomegaly, including left atrial and ventricular enlargement, is present in class B2. Class C includes animals with clinical and radiographic signs of left- or right-sided CHF, and class D includes animals in refractory end-stage CHF to standard treatment. The diagnosis of PDA was made on color Doppler echocardiography confirming the visualization of the PDA and direction of flow through the PDA, and only left-to-right shunt PDA was included in the PDA group (29). A diagnosis of PS was made by conventional echocardiographic criteria for PS in dogs, including obstructive abnormalities of the right outflow track, pulmonic valve, and pulmonary trunk (30). A diagnosis of HAC was made according to the clinical signs, serum biochemical analyses, and adrenocorticotropic hormone (ACTH) stimulation test consistent with HAC (31). Only dogs with LVH secondary to non-cardiac diseases were included in the LVH group. HAC is one of the major diseases that can affect cardiovascular abnormalities in dogs (32), and the LVH group in this study had only concurrent HAC. LVH was defined as regional or generalized concentric cardiomyopathy with an end-diastolic left ventricular posterior wall thickness (LVPWd) ≧7 mm or an end-diastolic interventricular septum thickness (IVSd) ≧6 mm in dogs with a body weight of <5 kg, and with ≧8 mm of the LVPWd or ≧7 mm of the IVSd in dogs with a body weight of 5–11 kg (29).

According to the cardiac hypertrophy type, dogs with heart diseases were divided into two groups to evaluate the galectin-3 levels. The eccentric cardiomyopathy group included dogs with volume overload heart diseases such as MMVD and PDA with normalized LV internal diameter at end-diastole (LVIDdn) ≥1.7 showing increased LV cavity or a left atrial-to-aortic root diameter ratio >1.6 based on the ACVIM criteria for LV and left atrial enlargement, respectively (28). The concentric cardiomyopathy group included dogs with pressure overload cardiac diseases, such as PS and SAS or dogs with LVH induced by non-cardiac causes, such as HAC (32). A diagnosis of SAS was made according to the stenotic area of the left ventricular outflow tract below the aortic valves from the left apical view by conventional echocardiographic criteria for SAS in dogs (33).

### Sample Collection

Stored serum was retrieved from the archived serum samples of the dogs, whose medical data were evaluated retrospectively. The archived serum samples were collected upon admission via jugular venipuncture into serum-separating tubes (BD Vacutainer^®^, USA) after an overnight fast. After 30 min of standing time, the tube was centrifuged at 1,500 × *g* for 15 min. The supernatant liquid, as serum, was stored frozen at −80°C until used.

### Measurement of Serum Galectin-3 Concentration

Enzyme-linked immunosorbent assay (ELISA) was performed to evaluate the serum galectin-3 concentration using a canine galectin-3 competitive ELISA kit (Dog antigen galectin-3 ELISA kit, BlueGene Biotech, Shanghai, China, Cat. No. E08G0052). The ELISA was performed following the instructions of the manufacturer. Briefly, kit components and serum samples were kept at room temperature (20–25°C) before use. Then, 100 μl of standard or sample was added to the appropriate wells. The same amount of phosphate-buffered saline (PBS, pH 7.0–7.2) was placed in the blank control well. Then, 50 μl of the conjugate was placed to each well except for the blank control and mixed wells. The microplate was covered and incubated at 37°C for 1 h, followed by five manual washes. Then, 50 μl of substrates A and B was placed in each well. The plate was incubated at 37°C for 15 min. After adding 50 μl of stop solution to each well, the optical density was analyzed at 450 nm using a microplate reader (SpectraMax 340, Molecular Device Co., Sunnyvale, CA, USA) immediately. The serum galectin-3 was measured in duplicate.

### N-Terminal Pro-B-type Natriuretic Peptide Level Examination

Archived serum samples of healthy dogs and dogs with heart diseases were stored frozen at −80°C and transported at ambient temperature to a reference laboratory (IDEXX Laboratories Inc, Westbrook, ME, USA) to analyze the NT-proBNP concentration. The NT-proBNP levels were measured using a second-generation ELISA (Cardiopet Canine proBNP, IDEXX Laboratories Inc, Westbrook, ME, USA), which has been previously validated in dogs. The reference interval of NT-proBNP was <900 pmol/L (27).

### Conventional Echocardiography

Echocardiography was performed in conscious, non-sedated dogs, and in the right and left lateral recumbency in the healthy and cardiac disease groups. Conventional echocardiographic data were obtained from medical records and consisted of transthoracic 2D, M-mode, spectral, and color flow Doppler studies performed using transducer arrays of 3–8 MHz (EPIQ 7 Ultrasound system, Philips, WA, USA) with continuous electrocardiography monitoring. For each dog, M-mode measurements were obtained at the level of the papillary muscles from the right parasternal short-axis view. These measurements included fractional shortening (FS), IVSd, LVPWd, LV internal diameter at end-diastole (LVIDd), and end-systole (LVIDs), and ejection fraction (EF) derived from the Teichholz method in the diastole and systole. The LV end-diastolic volume (EDV) and end-systolic volume (ESV) were calculated as the EDV and ESV indices by dividing them by the body surface area of each dog, respectively. The relative wall thickness was calculated as the ratio of the sum of the IVSd and LVPWd to LVIDd (34, 35). LVIDdn and normalized LVIDs (LVIDsn) were calculated according to the following formulas: LVIDdn = LVIDd (cm)/[BW (kg)]^0.294^ and LVIDsn = LVIDs (cm)/[BW (kg)]^0.315^ (36). The aorta and left atrium were measured in bidimensional mode on the right parasternal short-axis view obtained at the aortic valve level in the early diastole. The transmitral flow was obtained using the pulsed-wave Doppler technique with the left apical four-chamber view from the peak early diastolic wave (E) and peak late diastolic wave (A). The mitral annular motion velocity was measured using the left apical four-chamber view by the pulsed tissue Doppler technique. The tissue Doppler-derived peak velocity of the systolic mitral annular motion (S′), the tissue Doppler-derived peak early diastolic velocity (E′), and the tissue Doppler-derived peak late diastolic velocity (A′) were measured.

The LV diastolic function was evaluated using the E/A ratio and E/E′ ratio, and the criteria for the evaluation of the LV diastolic function was as follows: normal diastolic function, 1.0 < E/A < 2.0 and E/E′ < 12; grade one diastolic dysfunction as delayed relaxation pattern, E/A <1.0; grade two diastolic dysfunction as pseudo-normal pattern, 1.0 < E/A <2.0 and E/E′ ≥ 12; grade three diastolic dysfunction as restrictive pattern, E/A > 2.0 (33).

Echocardiograms were recorded and analyzed according to the echocardiographic criteria (29) and the Echocardiography Committee of the Specialty of Cardiology, ACVIM (37).

### Statistical Analysis

Statistical analyses were performed using IBM SPSS Statistics software program version 20.0 (IBM Corp., Armonk, NY, USA). All continuous data are presented as the mean ± standard deviation. The serum galectin-3 levels were compared between groups using an independent *t*-test when the values were normally distributed. Otherwise, the Mann–Whitney *U*-test was used for variables without normal distribution. Pearson's correlation analysis was performed to evaluate the correlation between the galectin-3 levels vs. conventional echocardiographic indices and galectin-3 levels vs. NT-proBNP levels. Receiver-operating characteristic (ROC) curves were used to evaluate the galectin-3 concentration as a predictor of diastolic dysfunction and advanced diastolic dysfunction. Statistical significance was set at *p* < 0.05.

## Results

### Baseline Characteristics

Of the 119 dogs that were included in the ELISA analysis, 12 were excluded as outliers. A total of 107 dogs that met the inclusion criteria were classified into healthy (8 dogs), cardiac disease (26 dogs), and non-cardiac disease groups (73 dogs). The cardiac disease group included dogs with MMVD (*n* = 13), PDA (L–R; *n* = 6), PS (*n* = 2), HAC with LVH (*n* = 2), subaortic stenosis (SAS; *n* = 1), PDA (R–L; *n* = 1), and DCM and SAS (*n* = 1). The non-cardiac disease group was classified into urologic (15 dogs), neoplastic (9 dogs), neurologic (12 dogs), immune-mediated (11 dogs), endocrine (18 dogs), and dermatologic groups (8 dogs) according to the affected organs. The urologic group included dogs with chronic kidney disease (*n* = 11), cystitis (*n* = 1), urinary calculi (*n* = 2), and renal cyst (*n* = 1). The neoplastic group included dogs with mast cell tumor (*n* = 4), lymphoma (*n* = 3), and nasal adenocarcinoma (*n* = 2). The neurologic group included only 12 dogs with meningoencephalitis of unknown origin. The immune-mediated group included dogs with immune-mediated hemolytic anemia (*n* = 3), immune-mediated thrombocytopenia (*n* = 3), Evan's syndrome (*n* = 1), and immune-mediated polyarthritis (*n* = 4). The endocrine group included dogs with DM (*n* = 4), HAC (*n* = 6), hypoadrenocorticism (*n* = 3), hypothyroidism (*n* = 3), and hypoaldosteronism (*n* = 2). The dermatologic group included only eight dogs with atopic dermatitis (AD).

The archived serum samples of 26 dogs with heart diseases, 73 dogs with non-cardiac diseases, and eight healthy dogs were included in the ELISA analysis. The breed distribution of the cardiac disease group was as follows: Pomeranian (*n* = 7), Shih-tzu (*n* = 6), Maltese (*n* = 3), mixed (*n* = 2), Yorkshire terrier (*n* = 2), Chihuahua (*n* = 2), Golden retriever (*n* = 1), Pekingese (*n* = 1), Welsh corgi (*n* = 1), and Poodle (*n* = 1). The breeds of the non-cardiac disease group included Pomeranian (*n* = 2), Shih-tzu (*n* = 6), Maltese (*n* = 19), mixed (*n* = 8), Yorkshire terrier (*n* = 4), Chihuahua (*n* = 6), Golden retriever (*n* = 1), Pekingese (*n* = 2), Poodle (*n* = 11), Cocker spaniel (*n* = 3), Spitz (*n* = 4), Bichon frise (*n* = 1), Coton de tulear (*n* = 1), Dachshund (*n* = 1), Jindo (*n* = 1), Miniature pinscher (*n* = 1), Papillion (*n* = 1), and Schnauzer (*n* = 1), and the healthy group included only Beagles. The healthy, cardiac disease, and non-cardiac disease groups were aged 2.38 ± 0.52, 8.13 ± 4.94, and 8.09 ± 4.13 years, respectively. The age of the cardiac and non-cardiac disease groups was significantly higher than that of the healthy group (*p* < 0.001 and *p* < 0.001, respectively).

### Galectin-3 Concentration in Dogs With Various Diseases

**Figure 1:**
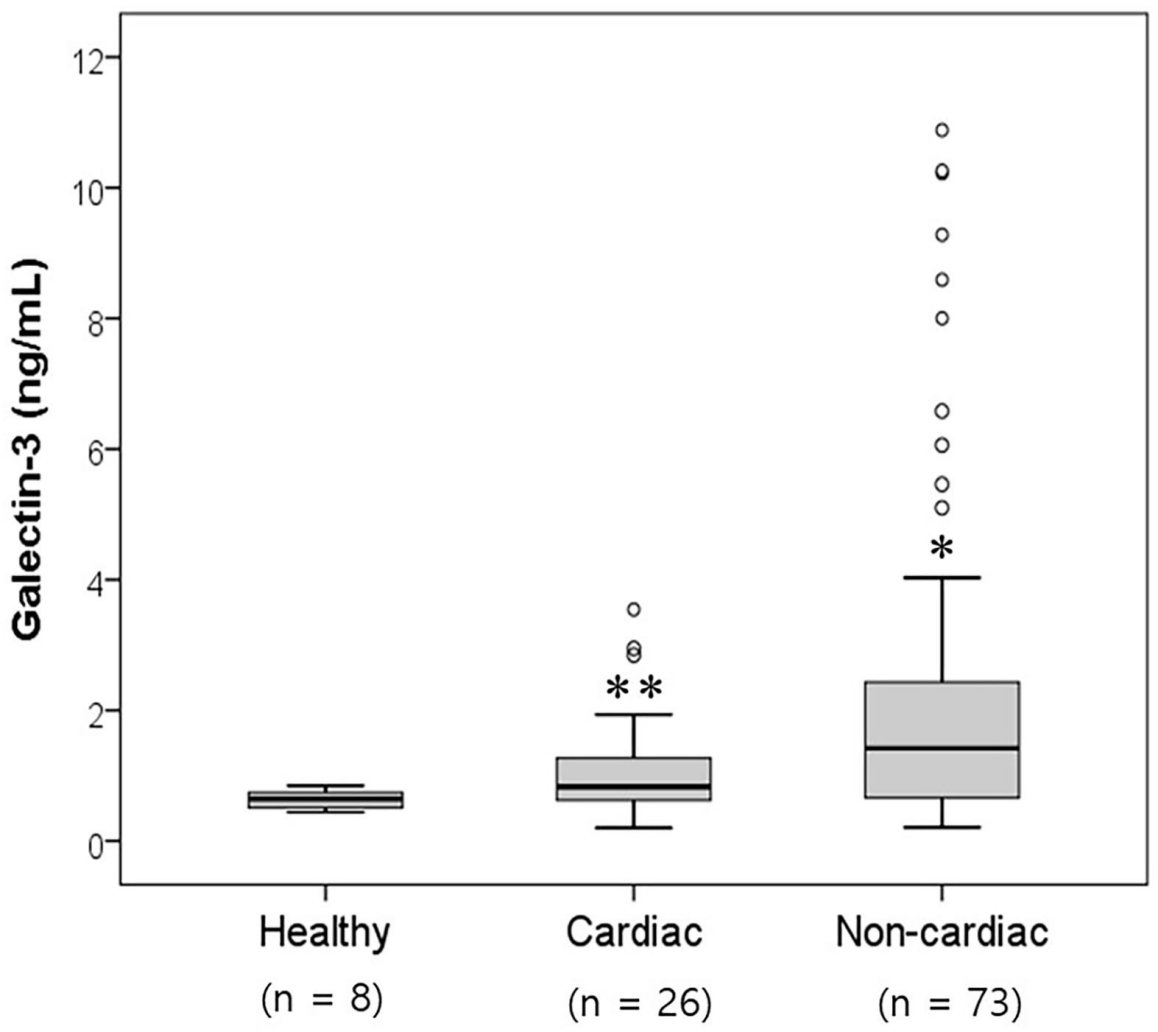
Comparison of the serum galectin-3 levels in healthy dogs and dogs with cardiac and non-cardiac diseases. The galectin-3 concentration was significantly higher in the cardiac and non-cardiac disease groups than in the healthy group (*p* < 0.05). Significantly different from the healthy group (*p < 0.05; **p < 0.01).

A total of 107 dogs were classified into three groups according to the prevalence of heart diseases (Table 1). An assessment of the galectin-3 levels in dogs according to the association with heart diseases is shown in Figure 1 and Table 2. Galectin-3 levels in the healthy, cardiac, and non-cardiac disease groups were 0.64 ± 0.15, 1.12 ± 0.83, and 2.27 ± 2.59 ng/ml, respectively. Dogs with heart diseases had significantly higher galectin-3 levels than healthy dogs (*p* = 0.009). Dogs with non-cardiac diseases had significantly elevated galectin-3 levels compared with healthy dogs (*p* = 0.016).

**Table 1:** Classification of 107 dogs according to the association of heart diseases.

**Group**	***n***
**Healthy group**	**8**
**Cardiac disease group**	**26**
MMVD	13
PDA (L to R)	6
PS	2
LVH	2
SAS	1
PDA (R to L)	1
DCM and SAS	1
**Non-cardiac disease group**	**73**
Urologic	15
Neoplastic	9
Neurologic	12
Immune-mediated	11
Endocrine	18
Dermatologic	8

*DCM, dilated cardiomyopathy; LVH, left ventricle hypertrophy; MMVD, myxomatous mitral valve disease; PDA, patent ductus arteriosus; PS, pulmonic stenosis; SAS, subaortic stenosis*.

**Table 2:** Galectin-3 levels in dogs according to the disease category.

**Variable**	**Healthy** ** (*n* = 8)**	**Cardiac** ** (*n* = 26)**	**Urologic** ** (*n* = 15)**	**Neoplastic** ** (*n* = 9)**	**Neurologic** ** (*n* = 12)**	**Immune-mediated** ** (*n* = 11)**	**Endocrine** ** (*n* = 18)**	**Dermatologic** ** (*n* = 8)**
Galectin-3 (ng/ml)	0.64 ± 0.15	1.12 ± 0.83^**^	1.62 ± 2.58	2.42 ± 3.34	2.35 ± 2.85	2.03 ± 2.12	2.48 ± 2.56^**^	3.05 ± 2.42^*^

* *Significantly different from the healthy group (p < 0.05)*.

** *Significantly different from the healthy group (p < 0.01)*.

The galectin-3 levels in the cardiac, urologic, neoplastic, neurologic, immune-mediated, endocrine, and dermatologic groups were 1.12 ± 0.83, 1.62 ± 2.58, 2.42 ± 3.34, 2.35 ± 2.85, 2.03 ± 2.12, 2.48 ± 2.56, and 3.05 ± 2.42 ng/ml, respectively (Table 2). The dogs with cardiac disease (1.12 ± 0.83 ng/ml) had significantly higher galectin-3 levels than the healthy dogs (0.64 ± 0.15 ng/ml, *p* = 0.009). Dogs with endocrine diseases (2.48 ± 2.56 ng/ml) had significantly elevated galectin-3 levels compared with healthy dogs (0.64 ± 0.15 ng/ml, *p* = 0.007). Dogs with dermatologic diseases (3.05 ± 2.42 ng/ml) had significantly higher galectin-3 levels than healthy dogs (0.64 ± 0.15 ng/ml, *p* = 0.026).

The serum galectin-3 levels were analyzed according to the heart disease type in the cardiac disease group. Among the 26 dogs with heart diseases, 13 dogs were included in the MMVD group, and six dogs were included in the PDA group. Dogs with other heart diseases except MMVD and PDA were excluded due to a small number for each diagnosis. The galectin-3 levels in the healthy, MMVD, and PDA groups were 0.64 ± 0.15, 0.91 ± 0.65, and 1.42 ± 0.9 ng/ml, respectively. The galectin-3 levels in the MMVD and PDA groups were also higher than those in the healthy group, but there was no significant difference (*p* > 0.05).

**Figure 2:**
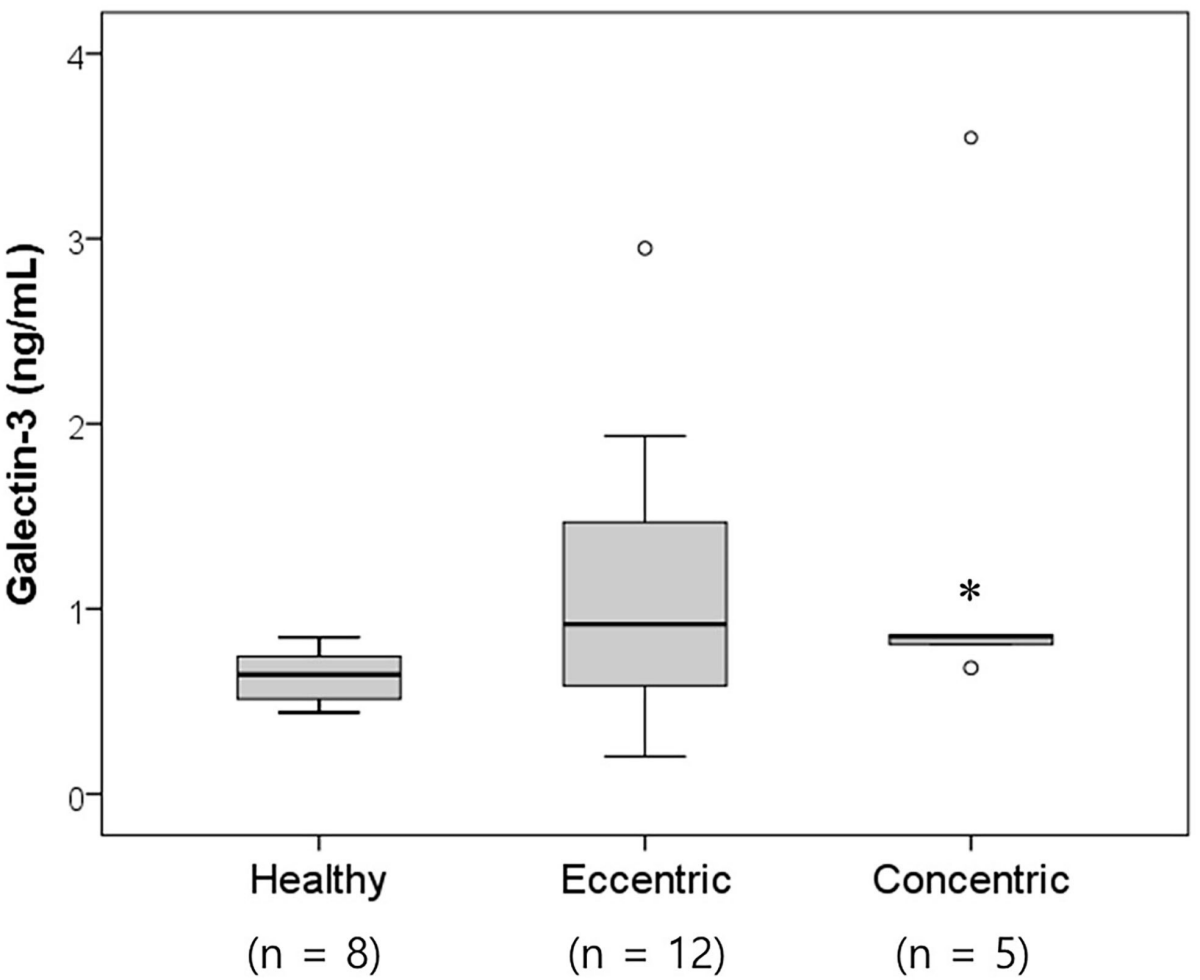
Box and whisker plots of the galectin-3 levels in healthy dogs and dogs with heart diseases according to the cardiac hypertrophy type. The concentric cardiomyopathy group had significantly increased galectin-3 levels compared with the healthy group (*p* = 0.028). Significantly different from the healthy group (*p < 0.05).

Twenty-six dogs with heart diseases were divided into two groups according to the cardiac hypertrophy type. The concentric cardiomyopathy group included the PS group (*n* = 2), LVH group (*n* = 2), and SAS group (*n* = 1). The eccentric cardiomyopathy group included MMVD dogs (*n* = 7) and PDA dogs (*n* = 5). Nine dogs not belonging to either the concentric or eccentric cardiomyopathy groups were excluded. The serum galectin-3 levels in the concentric and eccentric cardiomyopathy group were 1.35 ± 1.23 and 1.10 ± 0.77 ng/ml, respectively (Figure 2). Both the eccentric and concentric cardiomyopathy groups had an increased galectin-3 concentration compared with the healthy group (0.64 ± 0.15 ng/ml), but only the concentric cardiomyopathy group had significantly higher galectin-3 levels than the healthy group (*p* = 0.028).

Thirteen dogs with MMVD were classified by the ACVIM class. Six dogs belonged to ACVIM class B2, and the other seven dogs belonged to ACVIM class C. The dogs in ACVIM class B2 had higher galectin-3 levels (1.05 ± 0.90 ng/ml) than the healthy dogs (0.64 ± 0.15 ng/ml), but there was no significant difference between the two groups (*p* > 0.05). The dogs in ACVIM class C also had higher galectin-3 levels (0.80 ± 0.36 ng/ml) than the healthy dogs (0.64 ± 0.15 ng/ml) without significant difference (*p* > 0.05).

### Galectin-3 Concentration in Dogs With Cardiac Diseases

#### Correlation Between the Serum Galectin-3 Levels and Conventional Echocardiographic Parameters

The correlation between the galectin-3 levels and conventional echocardiographic indices was assessed in eight healthy dogs and 26 dogs with heart diseases (Table 3). Among the conventional echocardiographic indices, only E′/A′ had a significant correlation with the galectin-3 levels. E′/A′ had a significantly negative correlation with the galectin-3 levels (*r*^2^ = 0.117, *p* = 0.048).

**Table 3:** Bivariate correlation analysis of the conventional echocardiographic indices with galectin-3 concentration.

**Variables**	***r***	***p***
RWT	−0.038	0.831
LVIDdn	0.070	0.694
LVIDsn	0.067	0.705
EDVI	0.054	0.763
ESVI	0.017	0.924
FS	0.097	0.587
EF	0.008	0.965
LA/Ao	−0.068	0.702
PA/Ao	0.170	0.336
E/A	−0.194	0.270
E′/A′	−0.342	0.048^*^
E/E′	0.156	0.379

*A, transmitral peak late diastolic velocity; A′, tissue Doppler-derived peak late diastolic velocity; Ao, aorta; E, transmitral peak early diastolic velocity; E′, tissue Doppler-derived peak early diastolic velocity; EDVI, end-diastolic volume index; EF, ejection fraction; ESVI, end-systolic volume index; FS, fractional shortening; L, longitudinal; LA, left atrium; LVIDdn, normalized left ventricular internal diameter in diastole; LVIDsn, normalized left ventricular internal diameter in systole; PA, pulmonary artery; RWT, relative wall thickness*.

* *Values that have a significant correlation (p < 0.05)*.

**Figure 3:**
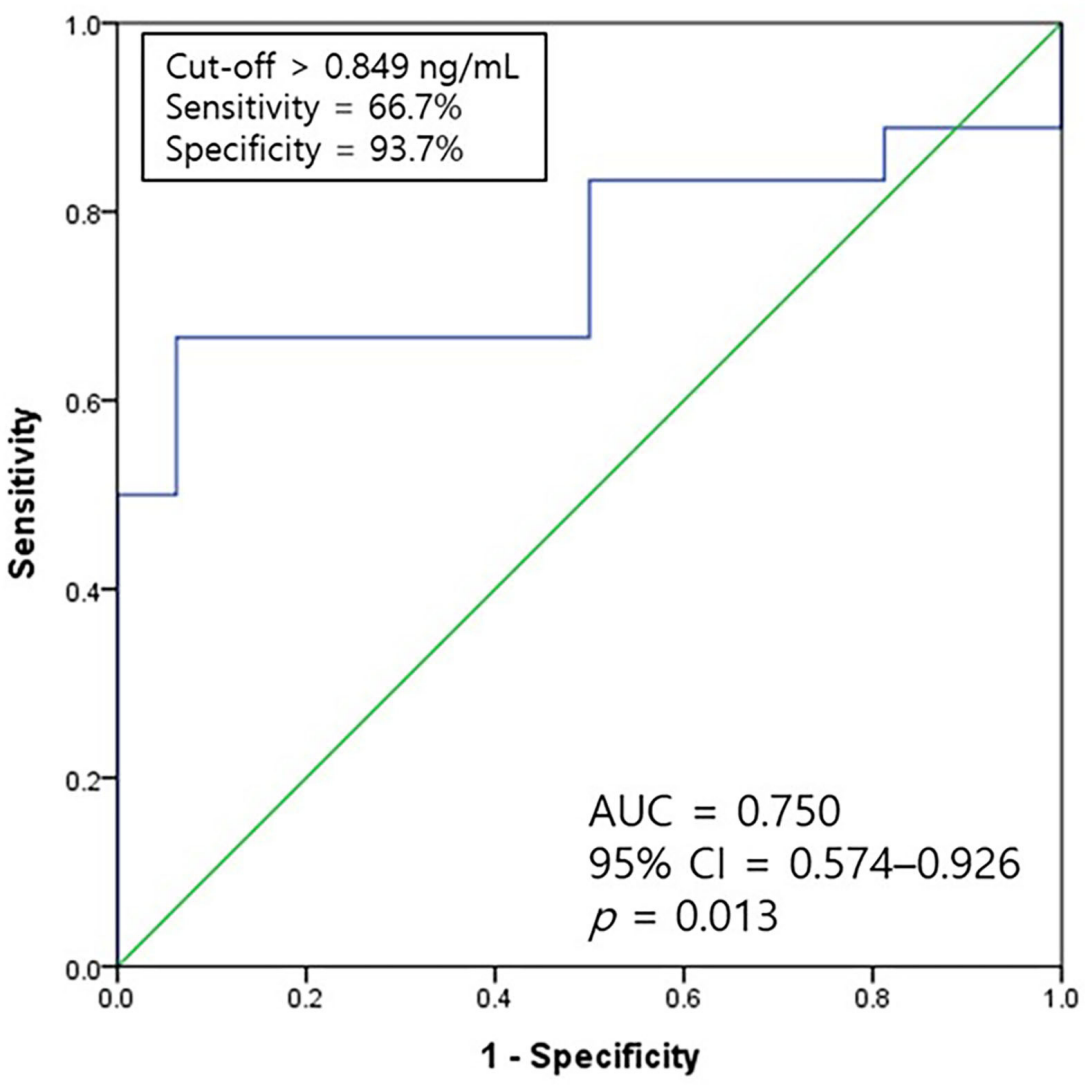
A ROC curve of galectin-3 for predicting the occurrence of diastolic dysfunction. The AUC value of galectin-3 was 0.750 with a sensitivity of 66.7% and specificity of 93.7% at a cutoff level of 0.849 ng/ml (*p* = 0.013). AUC, the area under the curve; CI, confidence interval; ROC, receiver-operating characteristic.

The ROC analysis was used to assess the cutoff values for the galectin-3 levels for predicting diastolic dysfunction among eight healthy dogs and 26 dogs with heart diseases. The ROC curve for diastolic dysfunction was evaluated (Figure 3). The area under the curve of the galectin-3 levels was 0.750 with a cutoff level of 0.849 ng/ml, 66.7% sensitivity, and 93.7% specificity (*p* = 0.013).

#### Correlation Between the Serum Galectin-3 and NT-proBNP Levels

The NT-proBNP levels were assessed in eight healthy dogs and 26 dogs with heart diseases. The NT-proBNP levels of healthy dogs were 365.63 ± 131.17 pmol/L and those of dogs with heart diseases were 3,407.54 ± 3,468.85 pmol/L. The cardiac disease group had significantly elevated NT-proBNP levels compared with the healthy group (*p* < 0.001).

**Figure 4:**
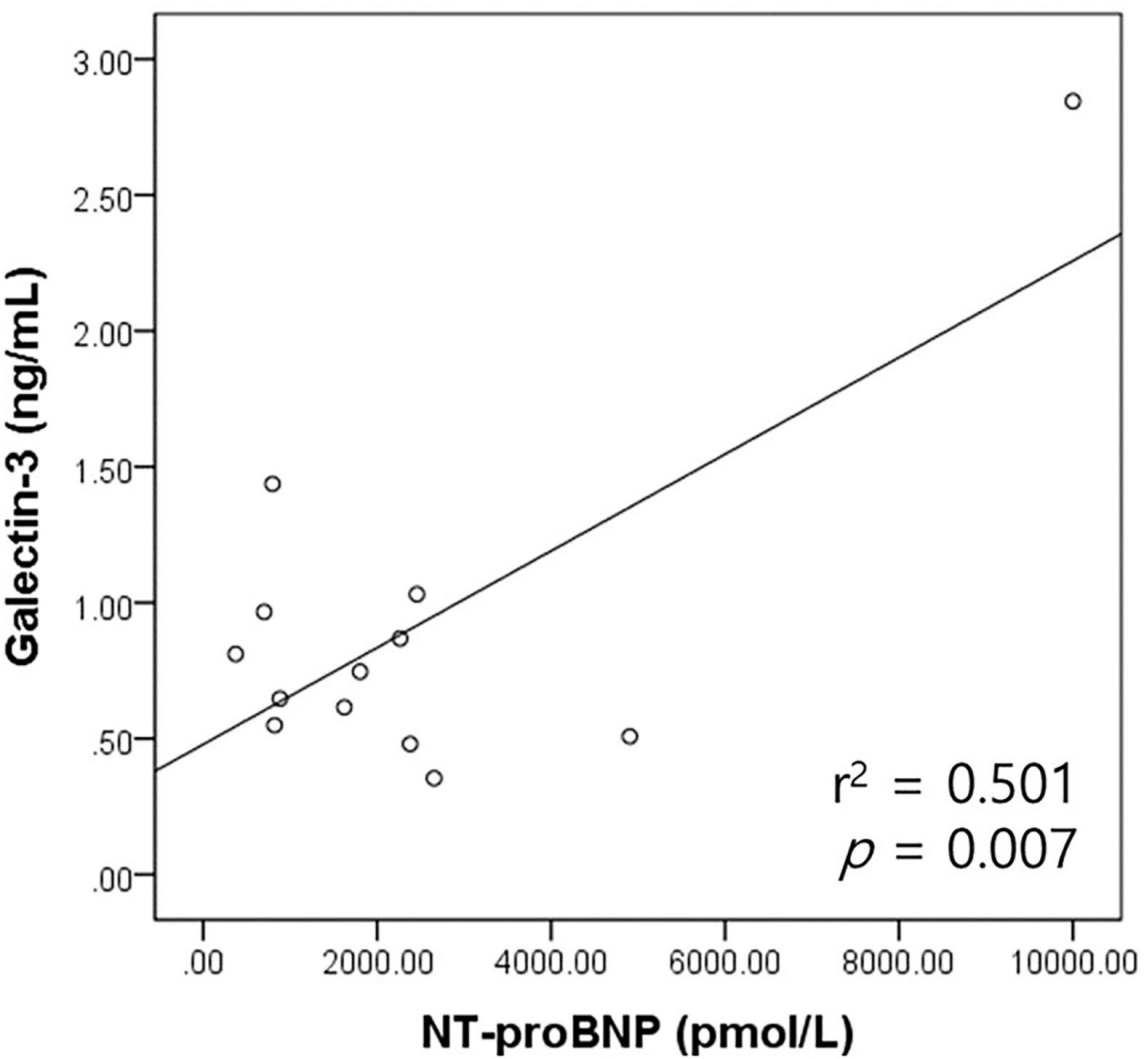
Scatter plot of the bivariate correlation analysis between the NT-proBNP and galectin-3 levels in dogs with MMVD. NT-proBNP levels had a significant positive correlation with galectin-3 levels in dogs with MMVD (*p* = 0.007). MMVD, myxomatous mitral valve disease; NT-proBNP, N-terminal pro-B-type natriuretic peptide.

The correlation between the galectin-3 and NT-proBNP levels was analyzed according to the heart disease type in the cardiac disease group. The correlation between the galectin-3 and NT-proBNP levels in the MMVD (*n* = 13) and PDA groups (*n* = 6) was assessed. There was a significant positive correlation between the galectin-3 concentration and NT-proBNP levels in the MMVD group (*r*^2^ = 0.501, *p* = 0.007; Figure 4). There was no significant correlation between the galectin-3 and NT-proBNP levels in dogs with PDA (*p* = 0.429).

## Discussion

The major finding of this study was the elevation of serum galectin-3 concentration in dogs with heart diseases, endocrine diseases, and dermatologic diseases compared with healthy dogs. Although research on the relationship between galectin-3 levels and diverse canine diseases remains scarce, several studies reported the association of galectin-3 in dogs with various diseases such as MMVD (7), oral melanoma (17), and mammary gland tumor (6). There has been no previous report evaluating galectin-3 in dogs with endocrine or inflammatory skin disease, but it is known that galectin-3 is related to specific endocrine or skin disease in humans (8, 38). Since the type of tumor in this study was different from the previously reported tumor, it was considered that the results of galectin-3 levels in dogs with neoplasia would be different from previous results. In cardiac diseases, galectin-3 has been widely known as cardiac biomarkers reflecting cardiac fibrosis in both humans (1) and dogs (39), and the results of this study supported the previous results that dogs affected by heart diseases had significantly higher galectin-3 levels than healthy dogs.

Cardiac fibrosis refers to several changes in the structure of interstitial collagen in the myocardium caused by a variety of cardiomyopathies (40). Cardiac fibrosis has a significant impact on the pathophysiology of cardiac systolic and diastolic dysfunction (20). In veterinary medicine, myocardial fibrosis has been noted in post-mortem findings of MMVD in dogs (41). Moreover, the severity of fibrosis was a risk factor for shorter survival times in dogs with CHF (42). The degree of myocardial fibrosis has been in the spotlight as a biomarker for determining the staging and prognosis in canine MMVD (43), so it would be clinically helpful if myocardial fibrosis could be assessed in dogs with heart diseases. Given these findings, it would be speculated that evaluation of galectin-3, which is associated with cardiac fibrosis (20), makes it possible to indirectly evaluate cardiac function and prognosis in dogs with heart diseases.

This study showed that dogs with heart diseases had significantly higher serum galectin-3 levels than healthy dogs. In human patients with heart diseases, including coronary heart disease, HF, and atrial fibrillation, increased circulating galectin-3 has been reported (15). Similar results have been reported in veterinary medicine, with the plasma galectin-3 concentration being reported to be increased in dogs with MMVD (7). Knockdown of galectin-3 in cardiac fibroblasts confirmed that galectin-3 played a crucial role in regulating cell survival, proliferation, and type I collagen synthesis *in vivo* (18). Studies on the correlation between galectin-3 and fibrosis in patients with various heart diseases, such as chronic Chagas disease cardiomyopathy (18) and aortic stenosis (AS) (44), have been reported in humans. Although the specific molecular mechanisms have not been identified, cardiac fibrosis and HF were induced by the stimulation of galectin-3 expression followed by promotion of protein kinase C-α (14). However, in this study, when categorizing dogs with heart diseases according to the cardiac diagnosis, higher galectin-3 levels were found in the MMVD and PDA groups compared with healthy dogs, but there was no significant difference between them. Although a statistical comparison of the heart disease type was considered invalid in this study, several factors might have influenced this result due to the nature of the retrospective study. In experimental studies, rats with induced myocardial infarction had reduced myocardial fibrosis after administration of angiotensin-converting enzyme inhibitors and spironolactone (45), indicating that circulating galectin-3 levels could be influenced by the type of medical treatment administrated. Treatment options for dogs with heart diseases varied in this study, so it might adversely affect the results of circulating galectin-3 levels. Moreover, the result was limited by the small number of dogs in each group classified according to the type of heart diseases among dogs with heart diseases. In addition to analyzing the effect of medications on galectin-3 concentration, further studies will be needed to find the association between survival times and the progression of diseases with galectin-3 levels in dogs with heart diseases on different treatments.

Concentric cardiomyopathy occurs by underlying heart diseases or non-cardiac causes. In this study, dogs with concentric cardiomyopathy induced by PS, SAS, or HAC were included in the concentric cardiomyopathy group. HAC is one of the representative non-cardiac diseases, which can cause LVH in dogs, and one report revealed that the prevalence of LVH among dogs with HAC was 68% (15/22) (32). In humans, HAC has been known to cause cardiovascular complications including both myocardial structural and functional abnormalities (46). HAC is associated with increased risk for cardiovascular diseases by excessive secretion of endogenous glucocorticoid, causing activation of the renin–angiotensin system and remodeling of LV, which is directly caused by cortisol-mediated activation of mineralocorticoid receptors in impaired myocardial tissues (47–49). This study demonstrated that dogs with concentric cardiomyopathy had significantly increased circulatory galectin-3 levels compared with healthy dogs. Similar findings have been reported previously in human AS patients with concentric hypertrophy (44). Galectin-3 is a protein that has been reported to be involved in the mechanism of LV remodeling (2). Galectin-3 is known as an important mediator, which has a significant pathologic effect on cardiac hypertrophy and HF (14). In patients with hypertrophic cardiomyopathy, galectin-3 expression levels were proportionally elevated according to the degree of hypertrophy (50). Concentric cardiomyopathy is known to be predisposed to the highest cardiovascular risk (51), so circulatory galectin-3 might be helpful for risk identification in dogs with cardiac remodeling. One experimental study showed that after injecting galectin-3 into the pericardial sac of rats, ventricular remodeling and cardiac dysfunction occurred (52). Taken together, galectin-3 might be strongly associated with cardiac remodeling and could predict patterns of geometric remodeling induced by cardiac interstitial fibrosis in dogs with heart diseases. Considering the results that galecin-3 expression of ACTH producing Cushing's disease was elevated in tumor tissues of humans (53), there is a possibility that the galectin-3 elevation of concentric cardiomyopathy group could be related to concurrent HAC. Although the association between HAC and galectin-3 in dogs has not been established yet, the effect of HAC on the galectin-3 levels should be considered as a limitation. Therefore, further large-scale studies on evaluating galectin-3 levels in dogs with LVH according to the underlying causes including heart disease or non-cardiac diseases will be needed.

Among the dogs with MMVD, the ACVIM B2 and C groups had increased serum galectin-3 concentrations, although non-significantly, compared with healthy dogs. Similar results were revealed previously that dogs with MMVD showed increased galectin-3 concentration without significant differences according to the ACVIM classification or severity of mitral regurgitation (7). In contrast, in humans with HF, the galectin-3 levels increased proportionally as the HF worsened (19). Moreover, human studies showed that the galectin-3 concentration was independently correlated with HF (1).

In this study, the galectin-3 concentration was found to be able to predict diastolic dysfunction. In agreement with this finding, diastolic parameters are associated with galectin-3 levels in dogs with diastolic dysfunction experimentally induced by thoracic aortic banding and humans with diastolic HF (54). Moreover, myocardial fibrosis is known as a pathophysiological factor that contributes to the systolic and diastolic dysfunction of the LV (20). Replacement of functional myocytes with collagen fibers caused by cardiac fibrosis is key to the progression of HF following cardiac dysfunction (55). Considering the result of increased galectin-3 levels in dogs with concentric cardiomyopathy, which tends to deteriorate the diastolic function compared with systolic function, galectin-3 is considered to have the ability to predict diastolic dysfunction induced by myocardial stiffness. Therefore, it is suggested that galectin-3 levels could be applied to predict for diastolic dysfunction in dogs with heart diseases.

Among the conventional echocardiographic indices, E′/A′ was found to have a significantly negative association with the galectin-3 levels in this study. E′/A′ is one of the parameters that indicates diastolic function and is assessed using the transmitral blood flow by a myocardial tissue Doppler (56). E′/A′ is known as an invasive parameter of LV stiffness and is correlated with LV filling pressures along with E/E′ (57). According to the results of this study, the ventricular diastolic function could be particularly affected by increased stiffness due to fibrosis of the heart, and galectin-3 could predict diastolic dysfunction. Therefore, the negative correlation found between E′/A′ and galectin-3 levels corresponds to the above results.

NT-proBNP, a biologically inert BNP, is a biomarker for evaluating cardiac function and predicting survival times in dogs (23, 24, 58) and humans (59). This study showed that galectin-3 had a significantly positive association with NT-proBNP levels in dogs with MMVD. Similar results were reported that circulating galectin-3 levels were correlated with BNP or NT-proBNP in humans (20). Although NT-proBNP is a biomarker mainly used for HF diagnosis, there is a limitation in that low BNP could not be completely ruled out in HF humans with preserved EF (60). Therefore, considering the role and prognostic impact of NT-proBNP, galectin-3 might be used clinically as a cardiac biomarker for assessing cardiac function and predicting prognosis along with NT-proBNP. Human studies reported that elevated galectin-3 levels increased the risk of developing HF and mortality when evaluated alone or in combination with BNP (20, 61). Galectin-3 is emerging as a prognostic biomarker in humans. Therefore, studies on the prognostic evaluation of galectin-3 are required for dogs with various heart diseases.

This study revealed that significantly higher galectin-3 concentrations were found in dogs with endocrine diseases when dogs were grouped according to the disease category. In a human study, the galectin-3 concentration was associated with the prevalence of DM, and elevated galectin-3 concentrations were found in humans with DM (8). It was revealed that galectin-3 was associated with the incidence of DM by acting on the inflammatory pathway that affects β-cell fibrosis and insulin secretion (8). Galectin-3 expression was also elevated in tumor tissues of humans with ACTH producing Cushing's disease (53). However, a few studies have examined the circulating galectin-3 concentration in animals with endocrine diseases. Although there have been no studies on the association between endocrine diseases and galectin-3 concentration in dogs, this study demonstrated significantly increased galectin-3 levels in dogs with endocrine diseases. These results support that the circulating galectin-3 concentration could be used as a diagnostic biomarker in dogs with endocrine diseases other than heart diseases. Further research is required on the galectin-3 levels in dogs with endocrine diseases.

Galectin-3 is involved by regulating the activity of a variety of cells such as fibroblasts and macrophages in various organs with the chronic inflammatory state (62). Galectin-3 plays a role in activating mast cells and basophils, and galectin-3 is involved in recruiting inflammatory cells in the skin (63, 64). Galectin-3 is highly expressed in various inflammatory and epithelial cells and can modify the induction and migration of inflammatory cells (38). Moreover, galectin-3 is known to contribute to the regulation of cellular homeostasis and promotion of cell adhesion (38). According to the results of this study, significantly higher galectin-3 concentrations were found in dogs with skin diseases diagnosed as AD. AD is a chronic pruritic inflammatory allergic skin condition that is genetically predisposed (65, 66). AD is relatively common in dogs and involved in immunoglobulin E (IgE)-mediated allergic reactions (67, 68). When evaluating the role of galectin-3 in a mouse model with AD induced by epicutaneous sensitization with ovalbumin, galectin-3-deficient mice had lower eosinophil infiltration, serum IgE levels, and thickening of the epidermis than those of galectin-3-expressing mice (69). Research on evaluating galectin-3 in dogs with AD or other inflammatory skin diseases is lacking, but the expression of galectin-3 was reported in dogs with squamous cell carcinoma of the skin (70). Given these findings, it could be speculated that galectin-3 could be a pro-inflammatory mediator in dogs with AD. Therefore, a large-scale prospective study on the effects of galectin-3 in dogs with AD is required.

There were several limitations to this study. The size of samples was relatively small. Second, serial monitoring of changes in galectin-3 was not conducted, so only a single measurement was performed. In addition, the association of galectin-3 concentration with clinical factors such as survival rates and prognosis could not be compared. Third, all dogs with heart diseases were treated with various treatment options, which might underestimate the concentration of circulating galectin-3 concentration. Last, the circulatory galectin-3 levels were not analyzed based on a comparison of their expression in cardiac tissues with histologically proven myocardial fibrosis. Despite these limitations, the present study demonstrates the possibility of using circulating galectin-3 concentration as a clinically useful diagnostic biomarker in dogs with heart diseases. It could be speculated that the elevation of circulatory galectin-3 levels was associated with cardiac fibrosis affecting cardiac remodeling and dysfunction in dogs with heart diseases.

In conclusion, this study has demonstrated that the circulating galectin-3 concentration increases in dogs with heart diseases as well as in dogs with endocrine and skin diseases. Moreover, galectin-3 levels might be a reliable predictor for cardiac remodeling in dogs with heart diseases. This study showed that galectin-3 could be used as a useful cardiac biomarker in diagnosing heart diseases and evaluating cardiac function in dogs. In addition to evaluating the potential role of circulating galectin-3 concentration as cardiac diagnostic biomarkers, further research is needed to assess myocardial fibrosis and galectin-3 expression in the heart tissues of dogs with heart diseases.

## Data Availability Statement

The original contributions generated for the study are included in the article/supplementary material, further inquiries can be directed to the corresponding author.

## Ethics Statement

Ethical review and approval was not required as serum samples and medical data were from a previous study. Informed consent was obtained from the present owners of the dogs for sample collection and any accompanying medical data.

## Author Contributions

G-WL was involved in writing the manuscript. G-WL and H-MP were involved in the study concept and design. M-HK, D-WS, W-BR, and H-MP were involved in the critical revision in analyzing the results. All authors read and approved the final manuscript.

## Conflict of Interest

The authors declare that the research was conducted in the absence of any commercial or financial relationships that could be construed as a potential conflict of interest.

## Publisher's Note

All claims expressed in this article are solely those of the authors and do not necessarily represent those of their affiliated organizations, or those of the publisher, the editors and the reviewers. Any product that may be evaluated in this article, or claim that may be made by its manufacturer, is not guaranteed or endorsed by the publisher.

## References

[1] Gehlken C Suthahar N Meijers WC de Boer RA. Galectin-3 in heart failure: an update of the last 3 years. Heart Fail Clin. (2018) 14:75–92. 10.1016/j.hfc.2017.08.009 29153203

[2] Vergaro G Del Franco A Giannoni A Prontera C Ripoli A Barison A. Galectin-3 and myocardial fibrosis in nonischemic dilated cardiomyopathy. Int J Cardiol. (2015) 184:96–100. 10.1016/j.ijcard.2015.02.008 25697876

[3] Chen SC Kuo PL. The role of galectin-3 in the kidneys. Int J Mol Sci. (2016) 17:565. 10.3390/ijms17040565 27089335 PMC4849021

[4] Ho MK Springer T. Mac-2, a novel 32,000 Mr mouse macrophage subpopulation-specific antigen defined by monoclonal antibodies. J Immunol. (1982) 128:1221–8. 6173426

[5] Suthahar N Meijers WC Silljé HH Ho JE Liu FT de Boer RA. Galectin-3 activation and inhibition in heart failure and cardiovascular disease: an update. Theranostics. (2018) 8:593–609. 10.7150/thno.22196 29344292 PMC5771079

[6] Ribeiro C Santos MS De Matos AJ Barros R Gaertner F Rutteman GR. Serum galectin-3 levels in dogs with metastatic and non-metastatic mammary tumors. In vivo. (2016) 30:13–6. 26709123

[7] Sakarin S Rungsipipat A Surachetpong S. Galectin-3 in cardiac muscle and circulation of dogs with degenerative mitral valve disease. J Vet Cardiol. (2016) 18:34–46. 10.1016/j.jvc.2015.10.007 26786977

[8] Vora A De Lemos JA Ayers C Grodin JL Lingvay I. Association of galectin-3 with diabetes mellitus in the dallas heart study. J Clin Endocrinol Metab. (2019) 104:4449–58. 10.1210/jc.2019-00398 31162551

[9] Cheng D Liang B Li Y. Serum galectin-3 as a potential marker for gastric cancer. Med Sci Mon Int Med J Exp Clin Res. (2015) 21:755–60. 10.12659/MSM.892386 25765552 PMC4370354

[10] Tang WW Shrestha K Shao Z Borowski AG Troughton RW Thomas JD. Usefulness of plasma galectin-3 levels in systolic heart failure to predict renal insufficiency and survival. Am J Cardiol. (2011) 108:385–90. 10.1016/j.amjcard.2011.03.056 21600537 PMC3137764

[11] Drechsler C Delgado G Wanner C Blouin K Pilz S Tomaschitz A. Galectin-3, renal function, and clinical outcomes: results from the LURIC and 4D studies. J Am Soc Nephrol. (2015) 26:2213–21. 10.1681/ASN.2014010093 25568176 PMC4552104

[12] Meijers WC Januzzi JL Defilippi C Adourian AS Shah SJ Van Veldhuisen DJ. Elevated plasma galectin-3 is associated with near-term rehospitalization in heart failure: a pooled analysis of 3 clinical trials. Am Heart J. (2014) 167:853–60.e4. 10.1016/j.ahj.2014.02.011 24890535

[13] van Kimmenade RR Januzzi JL Ellinor PT Sharma UC Bakker JA Low AF. Utility of amino-terminal pro-brain natriuretic peptide, galectin-3, and apelin for the evaluation of patients with acute heart failure. J Am Coll Cardiol. (2006) 48:1217–24. 10.1016/j.jacc.2006.03.061 16979009

[14] Song X Qian X Shen M Jiang R Wagner MB Ding G. Protein kinase C promotes cardiac fibrosis and heart failure by modulating galectin-3 expression. Biochim Biophys Acta Mol Cell Res. (2015) 1853:513–21. 10.1016/j.bbamcr.2014.12.001 25489662

[15] Dong R Zhang M Hu Q Zheng S Soh A Zheng Y. Galectin-3 as a novel biomarker for disease diagnosis and a target for therapy. Int J Mol Med. (2018) 41:599–614. 10.3892/ijmm.2017.3311 29207027 PMC5752178

[16] Hashmi S Al-Salam S. Galectin-3 is expressed in the myocardium very early post–myocardial infarction. Cardiovasc Pathol. (2015) 24:213–23. 10.1016/j.carpath.2014.12.001 25547609

[17] Vargas T Pulz L Ferro D Sobral R Venturini M Corrêa H. Galectin-3 expression correlates with post-surgical survival in canine oral melanomas. J Comp Pathol. (2019) 173:49–57. 10.1016/j.jcpa.2019.10.003 31812173

[18] de Freitas Souza BS Silva DN Carvalho RH de Almeida Sampaio GL Paredes BD França LA. Association of cardiac galectin-3 expression, myocarditis, and fibrosis in chronic chagas disease cardiomyopathy. Am J Patho. (2017) 187:1134–46. 10.1016/j.ajpath.2017.01.016 28322201

[19] Lok DJ Van Der Meer P Lipsic E Van Wijngaarden J Hillege HL van Veldhuisen DJ. Prognostic value of galectin-3, a novel marker of fibrosis, in patients with chronic heart failure: data from the DEAL-HF study. Clin Res Cardiol. (2010) 99:323–8. 10.1007/s00392-010-0125-y 20130888 PMC2858799

[20] Ho JE Liu C Lyass A Courchesne P Pencina MJ Vasan RS. Galectin-3, a marker of cardiac fibrosis, predicts incident heart failure in the community. J Am Coll Cardiol. (2012) 60:1249–56. 10.1016/j.jacc.2012.04.053 22939561 PMC3512095

[21] Fisher E. Heart disease in the dog. J Small Anim Pract. (1972) 13:553–60. 10.1111/j.1748-5827.1972.tb06884.x 5079570

[22] Yoshida Y Nakanishi K Daimon M Ishiwata J Sawada N Hirokawa M. Alteration of cardiac performance and serum B-type natriuretic peptide level in healthy aging. J Am Coll Cardiol. (2019) 74:1789–800. 10.1016/j.jacc.2019.07.080 31582139

[23] Brozaitiene J Mickuviene N Podlipskyte A Burkauskas J Buneviciuŝ R. Relationship and prognostic importance of thyroid hormone and N-terminal pro-B-type natriuretic peptide for patients after acute coronary syndromes: a longitudinal observational study. BMC Cardiovasc Disord. (2016) 16:1–12. 10.1186/s12872-016-0226-2 26892923 PMC4757967

[24] Pfister R Strack N Wielckens K Malchau G Erdmann E Schneider CA. The relationship and prognostic impact of low-T3 syndrome and NT-pro-BNP in cardiovascular patients. Int J Cardiol. (2010) 144:187–90. 10.1016/j.ijcard.2009.03.137 19423177

[25] Hiebert JB Vacek J Shah Z Rahman F Pierce JD. Use of speckle tracking to assess heart failure with preserved ejection fraction. J Cardiol. (2019) 74:397–402. 10.1016/j.jjcc.2019.06.004 31303358 PMC6764910

[26] Fox P Oyama M Hezzell M Rush J Nguyenba T DeFrancesco T. Relationship of plasma N-terminal pro-brain natriuretic peptide concentrations to heart failure classification and cause of respiratory distress in dogs using a 2nd generation ELISA assay. J Vet Intern Med. (2015) 29:171–9. 10.1111/jvim.12472 25308881 PMC4858067

[27] de Lima GV da Silveira Ferreira F. N-terminal-pro brain natriuretic peptides in dogs and cats: a technical and clinical review. Vet World. (2017) 10:1072–82. 10.14202/vetworld.2017.1072-1082 29062197 PMC5639106

[28] Keene BW Atkins CE Bonagura JD Fox PR Häggström J Fuentes VL. ACVIM consensus guidelines for the diagnosis and treatment of myxomatous mitral valve disease in dogs. J Vet Intern Med. (2019) 33:1127–40. 10.1111/jvim.15488 30974015 PMC6524084

[29] Boon JA. Veterinary Echocardiography. Chichester; West Sussex: JohnWiley & Sons (2011).

[30] Bussadori C Amberger C Le Bobinnec G Lombard C. Guidelines for the echocardiographic studies of suspected subaortic and pulmonic stenosis. J Vet Cardiol. (2000) 2:15–22. 10.1016/S1760-2734(06)70007-8 19081330

[31] Peterson ME. Diagnosis of hyperadrenocorticism in dogs. Clin Tech Small Anim Pract. (2007) 22:2–11. 10.1053/j.ctsap.2007.02.007 17542191

[32] Takano H Kokubu A Sugimoto K Sunahara H Aoki T Fujii Y. Left ventricular structural and functional abnormalities in dogs with hyperadrenocorticism. J Vet Cardiol. (2015) 17:173–81. 10.1016/j.jvc.2015.07.002 26319177

[33] De Madron E Chetboul V Bussadori C. Clinical Echocardiography of the Dog and Cat-E-Book. St. Louis, MO: Elsevier Health Sciences (2015).

[34] Dini FL Capozza P Donati F Simioniuc A Corciu AI Fontanive P. Patterns of left ventricular remodeling in chronic heart failure: prevalence and prognostic implications. Am Heart J. (2011) 161:1088–95. 10.1016/j.ahj.2011.03.027 21641355

[35] Suzuki R Matsumoto H Teshima T Koyama H. Clinical assessment of systolic myocardial deformations in dogs with chronic mitral valve insufficiency using two-dimensional speckle-tracking echocardiography. J Vet Cardiol. (2013) 15:41–9. 10.1016/j.jvc.2012.09.001 23429036

[36] Cornell CC Kittleson MD Torre PD Häggström J Lombard CW Pedersen HD. Allometric scaling of M-mode cardiac measurements in normal adult dogs. J Vet Intern Med. (2004) 18:311–21. 10.1111/j.1939-1676.2004.tb02551.x 15188817

[37] Thomas WP Gaber CE Jacobs GJ Kaplan PM Lombard CW Vet M. Recommendations for standards in transthoracic two-dimensional echocardiography in the dog and cat. J Vet Intern Med. (1993) 7:247–52. 10.1111/j.1939-1676.1993.tb01015.x 8246215

[38] Larsen L Chen HY Saegusa J Liu FT. Galectin-3 and the skin. J Dermatol Sci. (2011) 64:85–91. 10.1016/j.jdermsci.2011.07.008 21889881 PMC3192432

[39] Vichit P Rungsipipat A Surachetpong S. Changes of cardiac function in diabetic dogs. J Vet Cardiol. (2018) 20:438–50. 10.1016/j.jvc.2018.08.001 30217497

[40] Zhou Z Xu L Wang R Varga-Szemes A Durden JA Schoepf UJ. Quantification of doxorubicin-induced interstitial myocardial fibrosis in a beagle model using equilibrium contrast-enhanced computed tomography: a comparative study with cardiac magnetic resonance T1-mapping. Int J Cardiol. (2019) 281:150–5. 10.1016/j.ijcard.2019.01.021 30738608

[41] Falk T Ljungvall I Zois N Höglund K Olsen L Pedersen H. Cardiac troponin-I concentration, myocardial arteriosclerosis, and fibrosis in dogs with congestive heart failure because of myxomatous mitral valve disease. J Vet Intern Med. (2013) 27:500–6. 10.1111/jvim.12075 23551840

[42] Falk T Jönsson L Olsen LH Tarnow I Pedersen HD. Associations between cardiac pathology and clinical, echocardiographic and electrocardiographic findings in dogs with chronic congestive heart failure. Vet J. (2010) 185:68–74. 10.1016/j.tvjl.2010.04.016 20494597

[43] Hezzell MJ Falk T Olsen LH Boswood A Elliott J. Associations between N-terminal procollagen type III, fibrosis and echocardiographic indices in dogs that died due to myxomatous mitral valve disease. J Vet Cardiol. (2014) 16:257–64. 10.1016/j.jvc.2014.08.002 25292459

[44] Zhou K Zhou Y Zhao Y Tan C Yuan Z Li J. The relationship between galectin-3 and different patterns of ventricular geometry remodelling in aortic valve stenosis. Heart Lung Circ. (2016) 25:371–7. 10.1016/j.hlc.2015.08.021 26525848

[45] Zannad F Alla F Dousset B Perez A Pitt B. Limitation of excessive extracellular matrix turnover may contribute to survival benefit of spironolactone therapy in patients with congestive heart failure: insights from the randomized aldactone evaluation study (RALES). Circulation. (2000) 102:2700–6. 10.1161/01.CIR.102.22.2700 11094035

[46] Muiesan ML Lupia M Salvetti M Grigoletto C Sonino N Boscaro M. left ventricular structural and functional characteristics in cushing's syndrome. J Am Coll Cardiol. (2003) 41:2275–9. 10.1016/S0735-1097(03)00493-5 12821259

[47] Yiu KH Marsan NA Delgado V Biermasz NR Holman ER Smit JW. Increased myocardial fibrosis and left ventricular dysfunction in cushing's syndrome. Eur. J. Endocrinol. (2011) 166:27–34. 10.1530/EJE-11-0601 22004909

[48] Young MJ Lam EY Rickard AJ. Mineralocorticoid receptor activation and cardiac fibrosis. Clin. Sci. (2007) 112:467–75. 10.1042/CS20060275 17391102

[49] Mihailidou AS Loan Le TY Mardini M Funder JW. Glucocorticoids activate cardiac mineralocorticoid receptors during experimental myocardial infarction. Hypertension. (2009) 54:1306–12. 10.1161/HYPERTENSIONAHA.109.136242 19841288

[50] Tülüce SY Tülüce K Çil Z Emren SV Akyildiz ZI Ergene O. Galectin-3 levels in patients with hypertrophic cardiomyopathy and its relationship with left ventricular mass index and function. Anatol J Cardiol. (2016) 16:344. 10.1016/j.amjcard.2015.01.329 26488381 PMC5336784

[51] Lin YH Lin LY Wu YW Chien KL Lee CM Hsu RB. The relationship between serum galectin-3 and serum markers of cardiac extracellular matrix turnover in heart failure patients. Clin Chim Acta. (2009) 409:96–9. 10.1016/j.cca.2009.09.001 19747906

[52] Liu YH D'Ambrosio M Liao Td Peng H Rhaleb NE Sharma U. N-acetyl-seryl-aspartyl-lysyl-proline prevents cardiac remodeling and dysfunction induced by galectin-3, a mammalian adhesion/growth-regulatory lectin. Am J Physiol Heart Circ Physiol. (2009) 296:H404–12. 10.1152/ajpheart.00747.2008 19098114 PMC2643891

[53] Jin L Riss D Ruebel K Kajita S Scheithauer BW Horvath E. Galectin-3 expression in functioning and silent ACTH-producing adenomas. Endocr Pathol. (2005) 16:107–14. 10.1385/EP:16:2:107 16199895

[54] Wu CK Su MY Lee JK Chiang FT Hwang JJ Lin JL. Galectin-3 level and the severity of cardiac diastolic dysfunction using cellular and animal models and clinical indices. Sci Rep. (2015) 5:17007. 10.1038/srep17007 26582585 PMC4652206

[55] McCullough PA Olobatoke A Vanhecke TE. Galectin-3: a novel blood test for the evaluation and management of patients with heart failure. Rev Cardiovasc Med. (2011) 12:200–10. 10.3909/ricm0624 22249510

[56] Bamaiyi AJ Norton GR Peterson V Libhaber CD Sareli P Woodiwiss AJ. Limited contribution of left ventricular mass and remodelling to the impact of blood pressure on diastolic function in a community sample. J Hypertens. (2019) 37:1191–9. 10.1097/HJH.0000000000002051 31026244

[57] Nagueh SF Appleton CP Gillebert TC Marino PN Oh JK Smiseth OA. Recommendations for the evaluation of left ventricular diastolic function by echocardiography. Eur J Echocardiogr. (2009) 10:165–93. 10.1093/ejechocard/jep007 19270053

[58] Burchell RK Schoeman J. Advances in the understanding of the pathogenesis, progression and diagnosis of myxomatous mitral valve disease in dogs. J S Afr Vet Assoc. (2014) 85:e1–5. 10.4102/jsava.v85i1.1101 28235308

[59] Ponikowski P Voors A Anker S Bueno H Cleland J Coats A. Developed with the special contribution of the heart failure association (HFA) of the ESC. Eur J Heart Fail. (2016) 18:891–975. 10.1093/eurheartj/ehw128 27207191

[60] Huis A De Man F Van Rossum A Handoko M. How to diagnose heart failure with preserved ejection fraction: the value of invasive stress testing. Neth Heart J. (2016) 24:244–51. 10.1007/s12471-016-0811-0 26914917 PMC4796056

[61] Jagodzinski A Havulinna AS Appelbaum S Zeller T Jousilahti P Skytte-Johanssen S. Predictive value of galectin-3 for incident cardiovascular disease and heart failure in the population-based FINRISK 1997 cohort. Int J Cardiol. (2015) 192:33–9. 10.1016/j.ijcard.2015.05.040 25985013

[62] Slack R Mills R Mackinnon A. The therapeutic potential of galectin-3 inhibition in fibrotic disease. Int J Biochem Cell Biol. (2020) 130:105881. 10.1016/j.biocel.2020.105881 33181315

[63] Craig SS Krishnaswamy P Irani AMA Kepley CL Liu FT Schwartz LB. Immunoelectron microscopic localization of galectin-3, an IgE binding protein, in human mast cells and basophils. Anat Rec. (1995) 242:211–9. 10.1002/ar.1092420210 7668406

[64] Shi Z Meng Z Han Y Cao C Tan G Wang L. the involvement of galectin-3 in skin injury in systemic lupus erythematosus patients. Lupus. (2018) 27:621–7. 10.1177/0961203317736144 29058991

[65] Saridomichelakis MN Olivry T. An update on the treatment of canine atopic dermatitis. Vet J. (2016) 207:29–37. 10.1016/j.tvjl.2015.09.016 26586215

[66] Hensel P Santoro D Favrot C Hill P Griffin C. Canine atopic dermatitis: detailed guidelines for diagnosis and allergen identification. BMC Vet Res. (2015) 11:196. 10.1186/s12917-015-0515-5 26260508 PMC4531508

[67] Santoro D. Therapies in canine atopic dermatitis: an update. Vet Clin Small Ani Pract. (2019) 49:9–26. 10.1016/j.cvsm.2018.08.002 30262146

[68] Kang MH Kim HJ Jang HJ Park HM. sensitization rates of causative allergens for dogs with atopic dermatitis: detection of canine allergen-specific IgE. J Vet Sci. (2014) 15:545–50. 10.4142/jvs.2014.15.4.545 24962408 PMC4269597

[69] Saegusa J Hsu DK Chen HY Yu L Fermin A Fung MA. Galectin-3 is critical for the development of the allergic inflammatory response in a mouse model of atopic dermatitis. Am J Patho. (2009) 174:922–31. 10.2353/ajpath.2009.080500 19179612 PMC2665752

[70] Marques G Rocha L Vargas T Pulz L Huete G Cadrobbi K. Relationship of galectin-3 expression in canine cutaneous squamous cell carcinomas with histopathological grading and proliferation indices. J Comp Pathol. (2020) 178:16–21. 10.1016/j.jcpa.2020.06.004 32800103

